# Theoretical Analysis of Androstenone Adsorption on Chimpanzee and Gorilla Olfactory Receptors (G‐Protein): Statistical Physics Modeling, Molecular Docking Simulation, and Thermodynamic Investigation

**DOI:** 10.1002/prot.70166

**Published:** 2026-08-13

**Authors:** Ismahene Ben Khemis, Olfa Noureddine, Fatma Aouaini, Ghadeer Mohsen Albadrani, Ana Carolina Ferreira Piazzi Fuhr, Nizar Lefi, Salah Knani, GuilhermeLuiz Dotto, Abdelmottaleb Ben Lamine

**Affiliations:** ^1^ Laboratory of Quantum and Statistical Physics LR 18 ES 18, Faculty of Sciences of Monastir Monastir Tunisia; ^2^ Department of Physics, College of Science Princess NourahbintAbdulrahman University Riyadh Saudi Arabia; ^3^ Materials Science and Engineering Graduate Program Federal University of Pelotas (UFPel) Pelotas Brazil; ^4^ Department of Physics, College of Science Qassim University Buraidah Saudi Arabia; ^5^ Department of Physics, College of Science Northern Border University Arar Saudi Arabia; ^6^ Research Group on Adsorptive and Catalytic Process Engineering (ENGEPAC) Federal University of Santa Maria Santa Maria Brazil

**Keywords:** androstenone, chimpanzee and gorilla olfactory receptors, statistical physics theory

## Abstract

An advanced theory based on statistical physics was applied to microscopically investigate androstenone adsorption and its relationship with olfactory perception in chimpanzees and gorillas. For this purpose, dose–response curves of androstenone on olfactory receptors of chimpanzee OR7D4 and gorilla OR7D4 were analyzed by statistical physics. The two‐energy adsorption model fits the chimpanzee data well, and the gorilla data are best fit by the one‐energy adsorption model. The stereographic parameter analysis indicated that the studied pheromones were docked in a nonparallel manner in both species via a multimolecular mechanism. The analysis showed that the molecules interacted with different amino acid residues, resulting in distinct olfactory responses. The positive values of the molar adsorption energies indicate that an exothermic and physisorption process occurred in both olfactory systems. The docking analysis revealed that androstenone binds to the olfactory receptors of chimpanzees and gorillas through specific interactions with amino acid residues in the binding pockets. The docking results indicated distinct binding modes in each species, involving van der Waals, alkyl, and C–H interactions, which corroborated the energy trends predicted by the statistical physics model. These findings provided insights into the molecular mechanisms underlying the differential olfactory responses in chimpanzees and gorillas. Statistical physics was an excellent tool for evaluating and interpreting the interactions between androstenone and olfactory receptors.

**Abbreviations**
DNA: deoxyribonucleic acid
PCR: polymerase chain reaction

## 1 Introduction

Olfaction allows animals to interact with and learn about their surroundings [1, 2]. The odors emitted by animals, called pheromones, play a crucial role in several vital functions for animals and insects. These pheromones are essential for reproduction [1], for the mother–newborn relationship [3], and for protection by enabling the detection of predators [2]. The mammalian sense of smell can detect and distinguish among many chemical structures and characteristics [4]. Olfactory receptors, which are members of the superfamily of G protein‐coupled receptors and are composed of more than 300 amino acids, sense odorants [5, 6] and may be activated by a wide range of odorants or pheromones, from sublime to repugnant [7, 8, 9]. In this context, previous studies [10, 11, 12] have identified OR7D4 orthologs in eight species, including chimpanzee, gorilla, bonobo, mongoose lemur, colobus monkey, pygmy monkey, rhesus monkey, and orangutan. These species can develop sensitivity to the pheromone androstenone, which can be perceived as a pleasant (sweet, floral) or unpleasant (sweat, urine) odor. However, a portion of the population cannot detect it [13]. In this way, only two OR7D4 orthologs, chimpanzee OR7D4 and gorilla OR7D4, are studied in the present paper.

Olfactory response curves are often treated as adsorption isotherms, under the assumption that odorant detection arises from ligand‐receptor binding events whose probability of occurrence follows saturable adsorption‐type dynamics, meaning the response is described as the gradual saturation of receptor sites by odorant molecules as their concentration rises. In this context, the androstenone response on olfactory receptors of chimpanzees and gorillas was fit to an empirical adsorption model (Hill model) [12]. Over the years, traditional adsorption models, such as Hill and Beidler models, have been used to fit experimental olfactory response curves and to examine multiple olfactory systems [7, 8, 9]. However, these empirical models cannot fully describe the olfaction phenomenon and lack quantitative discussions of the model parameters, providing only the half‐saturation concentration. Additionally, the investigated olfactory systems of chimpanzees and gorillas have not been previously explored using molecular docking simulations or fitted via adsorption models based on statistical physics since it is assumed that the interactions between the pheromone and the olfactory receptors are merely reduced to adsorption mechanisms [14]. Hence, adsorption models, developed by Ben Lamine team using the statistical physics theory, may provide a rigorous theoretical framework for interpreting adsorption phenomena at the molecular level. For instance, multiple other fields of biology, chemistry, and physics, including gustatory perception [15], photoemissive dyes in photovoltaic devices [16], pollutant removal and water treatment [17], and hydrogen absorption for energy storage [18], may be investigated through the adjustment of experimental concentration‐response curves for biological systems or adsorption isotherms for adsorption systems using statistical physics models.

Viewed in this light, the primary goal of this paper is to deeply comprehend and theoretically characterize olfactory perception by modeling the androstenone response on the OR7D4 olfactory receptors of chimpanzees and gorillas. It treats the androstenone olfactory dose–response curves as adsorption equilibrium data, with the main objective of understanding, stereographically and energetically, the adsorption process in the chimpanzee and gorilla olfactory systems. Advanced statistical physics theory was used to study molecular and microscopic parameters, which may provide valuable information about the stereographic properties (number of docked molecules on one binding pocket, aggregation degree, anchorage manner and orientation, etc.) and the energetic properties (adsorption energy, adsorption type and nature, and types of interactions observed between the docked pheromone molecules and the receptor amino acid residues). The established models provide comprehensive information on both stereographic and energetic properties. The analysis of these parameters indirectly characterizes the mechanism of peripheral olfaction. To investigate the olfactory responsiveness of chimpanzees and gorillas to pheromones, molecular docking simulations focused on odorant‐receptor interactions and potential docking poses.

## 2 Methods

### 2.1 Odorant: Androstenone

The odorant used in this work is androstenone, a steroid pheromone related to testosterone [19, 20, 21]. Androstenone pheromone may be described as presenting a pleasant odor (sweet, floral) or an unpleasant odor (sweat, urine); however, it cannot be detected by a fraction of the population [13, 21]. The chemical structure of the pheromone is shown in Figure 1.

**FIGURE 1:**
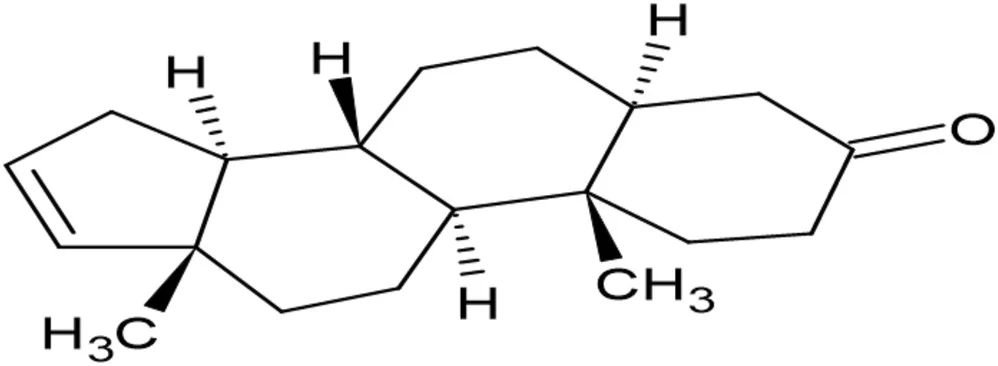
Chemical structure of androstenone.

### 2.2 Preparation of Chimpanzee and Gorilla Olfactory Receptors

#### 2.2.1 Plasmid Construction, Cloning, and Sequencing

The deoxyribonucleic acid (DNA) of a gorilla ( *Gorilla gorilla* , NG05251) and 11 chimpanzees ( *Pan troglodytes* , NG06939, NG03487, NG03489, NG03610, NG03622, NG03623, NG03641, NG03650, NG03656, NG03659, and NG03660) was obtained from the Coriell Cell Repository for the extraction of genomic DNA. Genomic DNA was extracted using the UltraCleanBloodSpin kit (MO BIO Laboratories).

OR7D4 was amplified from chimpanzee ( *P. troglodytes* ) and gorilla ( *G. gorilla* ) genomic DNA using polymerase chain reaction (PCR) and gene‐specific primers based on sequence data obtained from 3130 or 3730 genetic analyzers [12]. Partial sequences of apparent orthologs of these genes were obtained from primates, including chimpanzees and gorillas. The complete OR7D4 coding sequences in chimpanzees and gorillas were amplified using the following primers:

1. Primers forward: OR7D4_FW 5′‐ATGGAAGCAGAAAACCTTAC‐3′
2. Primers reverse: OR7D4_RV 5′‐ATGGAAGCAGAAAACCTTAC‐3′
  When the above primers failed to amplify, degenerate primers at reduced annealing temperatures were used:
3. FW5′‐ATGGAAGCAGAAAACYWTACAGAA‐3′
4. RV5′‐TCAYRGRCAAGAGKCKGYYCTGCTGAG‐3′
  If there was still no amplification with these primers, internal primers were used:
5. FW5′‐TTCCTGTCCATGTACCTGGT‐3′
6. RV5′‐GAGGCTGCCCTGCTGAGGAG‐3′

Amplifications of OR7D4 were performed from chimpanzee and gorilla genomic DNA using PCR. The products were sequenced, and the OR7D4 nucleotide and peptide sequences were aligned using *ClustalW* in MEGA4 [22]. Phylogenetic trees were constructed with branch support evaluated by a bootstrap of 1000 replications, showing a phylogeny congruent with that accepted for primates [23], confirming the orthology of the sequences obtained.

#### 2.2.2 Luciferase Assay and Data Analysis

Rho‐tagged olfactory receptors are transfected into the Hana3A cell line and hRTP1S, a short form of human RTP1. Adding Rho tags to olfactory receptors increased surface expression for chimpanzee OR7D4 and gorilla OR7D4 [24]. Cells are grown at 310 K in an incubator with 5% CO_2_. Stratagene's CRE‐luciferase assay was employed to assess receptor activation. Hana3A cells were cultured in 96‐well plates coated with poly‐d‐lysine (BioCoat from Becton Dickinson). Plasmid DNA containing the receptor and hRTP1S variants was transfected using Lipofectamine 2000. About 24 h after transfection, the medium was replaced with a chemically defined CD293 medium (Gibco), followed by a 30‐min incubation at 310 K with 5% CO_2_. Subsequently, the medium was replaced with a pheromone solution diluted in CD293, and the cells were incubated for 4 h at 310 K with 5% CO_2_. Luminescence was measured using a Perkin–Elmer Wallac Victor 1420 plate reader [12].

### 2.3 Dose‐Olfactory Response Curves

Data were analyzed using Microsoft Excel and GraphPad Prism 4. Normalized luciferase activity was calculated using the formula:

$$\begin{array}{c} \text{Normalized luciferase activity}\\ =\left[\mathrm{luc}\left(N\right)–\mathrm{luc}\left(\text{lowest}\right)\right]/\left[\mathrm{luc}\left(\text{highest}\right)–\mathrm{luc}\left(\text{lowest}\right)\right] \end{array}$$

where luc(*N*) represents the luminescence ratio of firefly luciferase to Renilla luciferase for a given well, luc(lowest) corresponds to the lowest firefly divided by Renilla luminescence ratio within a plate or set of plates, and luc(highest) corresponds to the highest firefly divided by Renilla luminescence ratio within that plate or set of plates. Luminescence assays are performed across a range of androstenone pheromone concentrations to generate dose–response curves. Experimental androstenone dose–response curves in chimpanzee and gorilla receptors were then acquired at room temperature, at 294 K. The dose–response curves for chimpanzee and gorilla olfactory receptors are experimentally recorded in arbitrary units (a.u.) and plotted against the logarithm of androstenone dose (concentration).

### 2.4 Statistical Physics Theory

The development and application of advanced models considered hypotheses derived from the statistical physics theory to enable the investigation of experimental dose‐olfactory response curves. The hypotheses considered are the following:

- The analysis of the adsorption mechanism in the olfactory system considered the transition of pheromone molecules from a dissolved state in mucus to a coupled state. Thus, for the application of statistical physics models, the grand canonical ensemble is selected as the most suitable statistical physics ensemble to take into account the variable number of pheromone molecules by adding a variable chemical potential during the docking process on chimpanzee OR7D4 and gorilla OR7D4 [25, 26].
- Mutual interactions between pheromone molecules are disregarded [26]. Due to the low concentration of odorous molecules in the olfactory systems studied, treating the coupled molecules as an ideal gas is possible [25, 26].
- Mucus is treated as an aqueous solution [25, 26].
- Only the translational and vibrational degrees of freedom of the molecules were considered [25, 26]. The electronic and rotational degrees of freedom were ignored because the former cannot be thermally excited, and the latter is hindered by mucus [25, 26, 27, 28].
- The concentration of the tested pheromone is low enough to ignore the interaction between the pheromone molecules and mucus. According to the Van't Hoff law for a diluted solution, the mucus is treated as an ideal solution, and the pheromone molecules in solution are treated as ideal gases [29]. Finally, it is supposed that the stimulation does not impoverish the mucus in any equilibrium state. This implies that this adsorption mechanism may be investigated using the grand canonical ensemble [29].

Based on these considerations, it is possible to develop an adsorption mechanism in the olfactory system. In the single adsorption energy model, a variable number of pheromone molecules are assumed to be anchored in an identical and independent binding pocket characterized by an adsorption energy $\varepsilon_{1}$. The two adsorption energy model assumes that pheromone molecules are anchored in two binding pockets, characterized by different adsorption energies, $\varepsilon_{1}$ and $\varepsilon_{2}$, respectively.

Each olfactory receptor binding pocket type in the analyzed mechanism can accommodate an average number of anchored molecules $\left(n_{i}\right)$ and presents an average olfactory response at saturation $R_{{\mathrm{sat}}_{i}}$ $\left(i=1,2\right)$. In the statistical physics models for the system under analysis, the grand canonical set is considered, in which the expression of the grand canonical partition function for each type of olfactory receptor binding pocket $Z_{i}$ is described by Equation (1) [27, 28, 29, 30, 31].

(1) $$Z_{i}=\sum_{N_{i}}e^{-\beta \left(\mu +\varepsilon_{i}\right)N_{i}}=1+e^{-\beta \left(\varepsilon_{i}+\mu \right)}$$

where $\varepsilon_{i}$ is the adsorption energy of each type of olfactory receptor binding pockets (i=1,2), μ is the chemical potential of olfactory receptor binding pockets, $N_{i}$ is the occupation number state of the olfactory receptor binding pockets (i.e., each olfactory receptor binding pocket may be vacant ($N_{i}=0$) or occupied ($N_{i}=1$)); and β is the factor of Boltzmann.

The occupancy number of each type of olfactory receptor binding pocket $\left(N_{0_{i}}\right)$ is described by Equation (2), where $D_{i}$ is the density of each type of olfactory receptor binding pocket (i=1,2) [28, 29].

(2) $$N_{0_{i}}=\frac{1}{\beta }\frac{\partial \ln{\left(Z_{i}\right)}^{D_{i}}}{\partial \mu }$$

The Polanyi expression, which relates the concentration of pheromone molecules to the adsorption energy of the type iεmi=εini olfactory receptor binding pocket, where $n_{i}$ is the number of androstenone molecules anchored in a type i binding pocket, which can be used when thermodynamic equilibrium is reached, is expressed by Equation (3) [32], where $C_{s}$ is the androstenone solubility in water. Additionally, upon reaching thermodynamic equilibrium, the μ of the molecules can be described according to Equation (4) [26].

(3) $$\frac{C}{C_{s}}=e^{-\beta \varepsilon_{m}}$$

(4) $$\mu =\frac{1}{\beta }\ln\left(\frac{\mathrm{NV}}{C_{s}}\right)=\frac{1}{\beta }\ln\left(\frac{C}{C_{s}}\right)$$

Considering the thermodynamic equilibrium conditions and the expression of the occupancy number of each type of olfactory receptor binding pocket $N_{0_{i}}$. Equation (2) can be rewritten according to Equation (5).

(5) $$N_{0_{i}}=\frac{D_{i}}{1+{\left(e^{\beta \varepsilon_{m}}\frac{C}{C_{s}}\right)}^{n_{i}}}$$

Under the condition of half‐saturation, the $N_{0_{i}}$ equals half the number of effectively occupied receptor sites or $D_{i}$. Thus, the half‐saturation concentration, $C_{i}$, can be expressed by Equation (6). In this case, the $N_{0_{i}}$ can be expressed as a function of the $C_{i}$, as shown in Equation (7).

(6) $$\frac{D_{i}}{2}=\frac{D_{i}}{1+\left(e^{\beta \varepsilon_{i}}\frac{C_{i}}{C_{s}}\right)}$$

(7) $$N_{0_{i}}=\frac{D_{i}}{1+{\left(\frac{C_{i}}{C}\right)}^{n_{i}}}$$

$C_{i}$ can be expressed as a function of the molar adsorption energy ($\Delta E_{i}$) of each type of olfactory receptor binding pocket according to Equation (8), where R is the ideal gas constant, T is the temperature, and $k_{B}$ is the Boltzmann constant.

(8) $$C_{i}=C_{s}e^{-\beta \varepsilon_{i}}=C_{s}e^{-\frac{\varepsilon_{i}}{k_{B}T}}=C_{s}e^{-\frac{\Delta E_{i}}{\mathrm{RT}}}$$

At thermodynamic equilibrium, the olfactory response of each type of binding pocket ($R_{\text{olfactoryi}}$) is proportional to the $N_{0_{i}}$ and can be described by Equation (9), where $\alpha_{i}$ is the coefficient of the transduction relative to $N_{0_{i}}$ (i = 1, 2), $R_{{\mathrm{sat}}_{i}}$ is the olfactory response at saturation relative to each type of binding pocket (i = 1, 2) [25, 27, 28, 29, 33].

(9) $$R_{\text{olfactoryi}}=\alpha_{i}N_{0_{i}}=\frac{\alpha_{i}D_{i}}{1+{\left(\frac{C_{i}}{C}\right)}^{n_{i}}}=\frac{R_{{\mathrm{sat}}_{i}}}{1+{\left(\frac{C_{i}}{C}\right)}^{n_{i}}}$$

After expressing $R_{\text{olfactoryi}}$ as a function of $\alpha_{i}$ and $D_{i}$ and concerning the limiting concentration ($C_{\mathrm{th}}$), the expression of the $R_{\text{olfactoryi}}$ as a function of the concentration of pheromone molecules can be defined by the following system presented in Equation (10):

(10) $$R_{\text{olfactoryi}}=\left\{\begin{array}{c} 0,\;C<C_{\mathrm{th}}\\ \frac{R_{{\mathrm{sat}}_{i}}}{1+{\left(\frac{C_{i}}{C}\right)}^{n_{i}}},C\geq C_{\mathrm{th}} \end{array}\right.$$

The grand canonical partition functions and the total olfactory response equations relative to the single adsorption energy model and the two adsorption energy model are presented in Table 1. The model performance was analyzed using the correlation coefficient ($R^{2}$) and regression mean square error (RMSE).

**TABLE 1:** The grand‐canonical partition functions and analytical equations for the two statistical physics models.

Models	Grand canonical partition functions	Model equations
One adsorption energy model	Grand canonical partition function for one binding pocket $Z_{1}=\sum_{N_{i=0,1}}e^{-\beta \left(\mu_{1}+\varepsilon_{1}\right)N_{i}}=1+e^{-\beta \left(\mu_{1}+\varepsilon_{1}\right)}$	$R_{\text{olfactory}}=\frac{R_{{\mathrm{sat}}_{1}}}{1+{\left(\frac{C_{1}}{C}\right)}^{n_{1}}}$
Two adsorption energy model	Grand canonical partition functions for one binding pocket $Z_{1}=\sum_{N_{i=0,1}}e^{-\beta \left(\mu_{1}+\varepsilon_{1}\right)N_{i}}=1+e^{-\beta \left(\mu_{1}+\varepsilon_{1}\right)}$ $Z_{2}=\sum_{N_{i=0,1}}e^{-\beta \left(\mu_{2}+\varepsilon_{2}\right)N_{i}}=1+e^{-\beta \left(\mu_{2}+\varepsilon_{2}\right)}$	$R_{\text{olfactory}}=\frac{R_{{\mathrm{sat}}_{1}}}{1+{\left(\frac{C_{1}}{C}\right)}^{n_{1}}}+\frac{R_{{\mathrm{sat}}_{2}}}{1+{\left(\frac{C_{2}}{C}\right)}^{n_{2}}}$

## 3 Results and Discussion

### 3.1 Results

#### 3.1.1 Experimental Curves

The experimental curves of the olfactory dose response of androstenone on chimpanzee OR7D4 and gorilla OR7D4 at room temperature (~294 K) are represented in Figure 2 [12]. The experimental data revealed two distinct olfactory responses of OR7D4 to the pheromone androstenone in chimpanzees ( *P. troglodytes* ) and gorillas ( *G. gorilla* ). The chimpanzee olfactory response ranged from 3 × 10^−9^ to 3 × 10^−5^mol L^−1^, while the gorilla olfactory response ranged from 10^−7^ to 3 × 10^−5^mol L^−1^.

**FIGURE 2:**
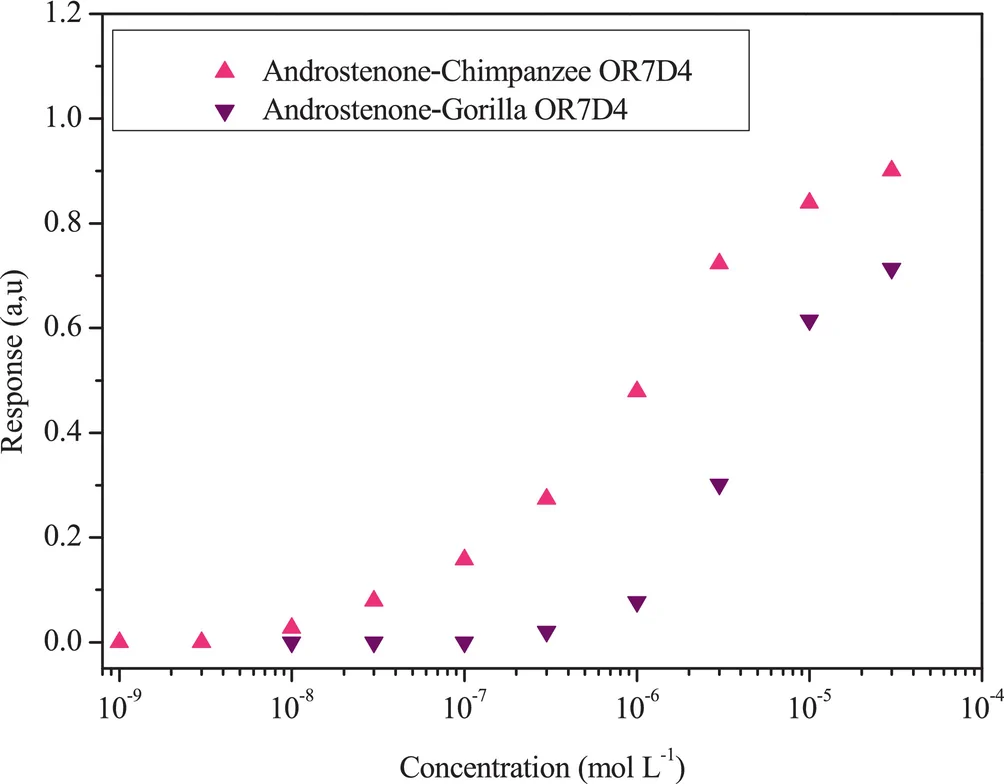
Experimental concentration‐response curves of chimpanzee and gorilla olfactory receptors activated by androstenone [12].

It is clear that the olfactory responses of androstenone pheromone molecules on chimpanzee OR7D4 and gorilla OR7D4 exhibit sigmoid and hyperbolic forms, with different saturation levels and threshold concentrations. The difference in the response maxima of the two olfactory systems shows that the interaction types between these two olfactory receptors and the tested pheromone play an essential role in the intensity difference.

Based on the set of experiments carried out by Zhuang et al. [12], a numerical simulation using advanced modeling and statistical physics theory was employed to understand the molecular‐level mechanism underlying the olfactory response of chimpanzees and gorillas activated by the pheromone androstenone.

#### 3.1.2 Experimental Data Modeling: Selection of the Appropriate Model

The olfactory dose–response curves of androstenone on OR7D4 olfactory receptors of chimpanzees and gorillas were fitted using two statistical physics models. The best model was selected through a performance analysis using the correlation coefficient ($R^{2}$), the regression mean squared error (RMSE), and the physical analysis of the adjusted parameters for each model analyzed [34, 35, 36].

The model performance results indicated that the $R^{2}$ and RMSE values obtained by each model evaluated for the system under analysis ranged from 0.99771 to 0.99989 and from 0.12782 to 0.79095, respectively (Table 2). Furthermore, the two adsorption energy models presented the best statistical performance, exhibiting the highest $R^{2}$ value and the lowest RMSE value for the chimpanzee olfactory system. In contrast, the one‐energy adsorption model outperformed the gorilla's olfactory system.

**TABLE 2:** Values $R^{2}$ and the RMSE for the chimpanzee and the Gorilla olfactory systems.

Adsorption models	Chimpanzee OR7D4	Gorilla OR7D4
$R^{2}$		
One adsorption energy model	0.99771	0.99989
Two adsorption energy model	0.99919	0.99986
RMSE		
One adsorption energy model	0.79095	0.12782
Two adsorption energy model	0.39575	0.36392

The analysis of the estimated parameters for each model showed that the model with two adsorption energies can microscopically interpret the presumed adsorption process underlying the olfactory perception of the pheromone androstenone by the chimpanzee OR7D4 receptor. In contrast, the model with a single adsorption energy is better suited for interpreting the adsorption process of androstenone molecules on gorilla OR7D4. The estimated parameters for each model with the highest statistical performance and best interpretation of the process are presented in Table 3, and the results of fitting the experimental curves are represented in Figure 3.

**TABLE 3:** Physicochemical parameters relative to the one‐adsorption‐energy model and the two‐adsorption‐energy model.

Used models	Fitted parameters	Olfactory systems	
		Chimpanzee OR7D4	Gorilla OR7D4
One adsorption energy model	$R_{{\mathrm{sat}}_{1}}$ (a, u)	0.96	0.73
	$n_{1}$	0.78	1.62
	$C_{1}$ (mol L^−1^)	9.10 × 10^−7^	4.14 × 10^−6^
Two adsorption energy model	$R_{{\mathrm{sat}}_{1}}$ (a, u)	0.75	0.57
	$R_{{\mathrm{sat}}_{2}}$ (a, u)	0.15	0.17
	$n_{1}$	1.16	1.31
	$n_{2}$	1.34	1.50
	$C_{1}$ (mol L^−1^)	1.23 × 10^−6^	3.76 × 10^−6^
	$C_{2}$ (mol L^−1^)	3.60 × 10^−8^	3.07 × 10^−6^

**FIGURE 3:**
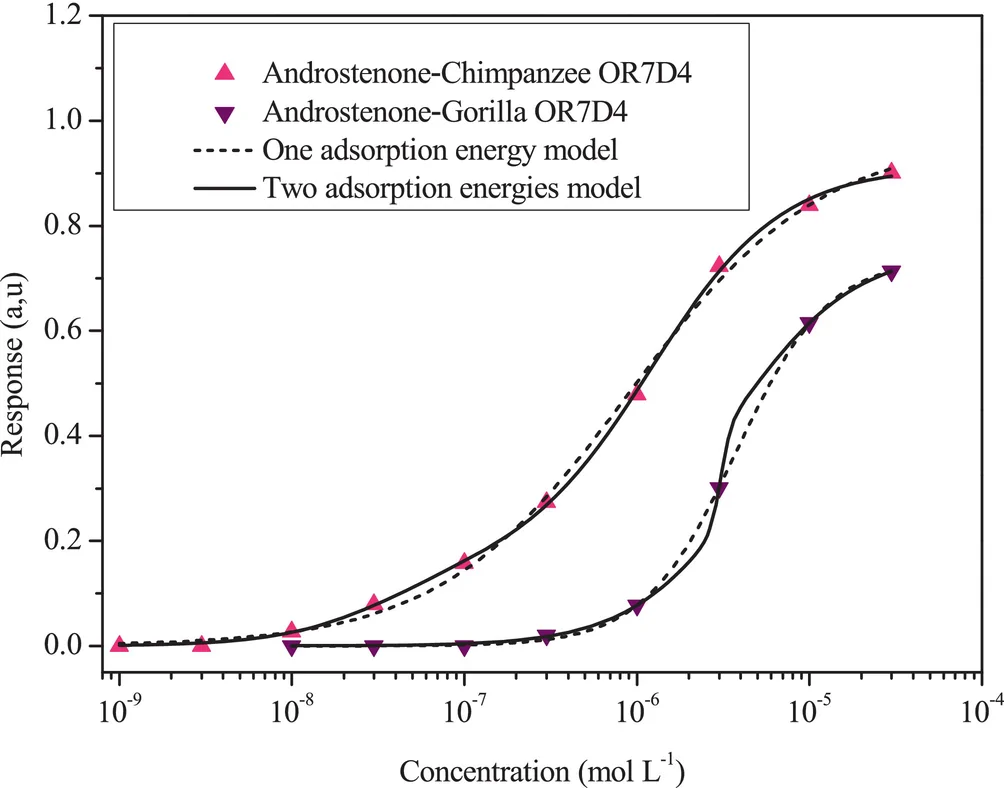
Adjusted experimental concentration‐response curves of androstenone pheromone on chimpanzee OR7D4 and gorilla OR7D4 with the applied models (illustrated by a short dash line for the one adsorption energy model and by a straight line for the two adsorption energy model).

The estimated physicochemical parameters of the two appropriate models can be classified into stereographic parameters ($n_{1}$, $n_{2}$, $R_{{\mathrm{sat}}_{1}}$, and $R_{{\mathrm{sat}}_{2}}$) and energetic parameters ($C_{1}$, $C_{2}$, $\Delta E_{1}$, and $\Delta E_{2}$).

### 3.2 Discussion

#### 3.2.1 Stereographic Analysis

##### 3.2.1.1 Number of Docked Androstenone Molecules on One Binding Pocket ($n_{1}$ and $n_{2}$)

Parameters $n_{1}$ and $n_{2}$ can describe the behavior of androstenone molecules in mucus, estimating the degree and mechanism of aggregation before adsorption and the behavior on the surface of the olfactory receptor after adsorption. Furthermore, these stereographic parameters indicate the interactions between the studied pheromone molecules and the binding pockets of chimpanzee and gorilla olfactory receptors, including their positions and orientations. Three cases of adsorption orientations can be distinguished based on the $n_{i}$ values (i = 1, 2) [27, 37].

- When $n_{i}$ < 0.5, the orientation of the molecules is parallel to the surfaces of the chimpanzee and gorilla OR7D4 receptors, resulting in a multianchor‐point adsorption system.
- When 0.5 < $n_{i}$ < 1, the molecules have a mixed orientation, both parallel and nonparallel to the surface. In this range, the adsorption system is classified as mixed.
- When $n_{i}$ > 1, the molecules are oriented nonparallel to the surface, and adsorption occurs in multimolecular docking [27, 37].

Analysis of the $n_{i}$ values (Table 3) reveals that the $n_{i}$ values for the chimpanzee and gorilla olfactory systems are greater than 1, ranging between 1 and 2 (1.16 < $n_{i}$ < 1.62). This trend indicates that androstenone pheromone molecules are anchored in the binding pockets of the studied olfactory receptors in a nonparallel orientation. In other words, a Type 1 or Type 2 binding pocket can be occupied by one or two androstenone molecules, with respective percentages x and (1−x). The following expression can determine the percentages of the two forms of interaction [38, 39].

- For androstenone molecules docked in the Type 1 binding pocket of the chimpanzee OR7D4 receptor, 84% of the pheromone molecules were docked as a single molecule per binding pocket. In comparison, 16% were docked as two aggregated molecules in a single binding pocket.
- For androstenone molecules anchored in the Type 2 binding pockets of the chimpanzee OR7D4 receptor, 66% of the molecules were adsorbed as single molecules per binding pocket. In comparison, 34% were adsorbed as two aggregated molecules on a single binding pocket.
- For androstenone molecules anchored on gorilla OR7D4 binding pockets, 38% of the molecules were adsorbed as single molecules per binding pocket. In comparison, 62% were adsorbed as two aggregated molecules on a single binding pocket.

The results obtained by $n_{i}$ show that androstenone molecules couple as monomers or dimers in the different binding pockets of the two olfactory systems. For example, two androstenone molecules can form a cluster via pi–pi interactions. The differences in degrees of aggregation are related to diverse interactions with the functional groups of amino acid residues that make up the binding pockets of the chimpanzee and gorilla OR7D4 receptors. Additionally, androstenone pheromone molecules dock and aggregate more efficiently in gorilla OR7D4 binding pockets compared to chimpanzee OR7D4 binding pockets.

##### 3.2.1.2 Olfactory Responses at Saturation ($R_{{\mathrm{sat}}_{1}}$ and $R_{{\mathrm{sat}}_{2}}$)

$R_{{\mathrm{sat}}_{1}}$ and $R_{{\mathrm{sat}}_{2}}$ provide valuable information on the adsorption processes underlying the olfactory perceptions of chimpanzees and gorillas. Furthermore, they allow a stereographic characterization of the first and most crucial stage of the two olfactory systems investigated. These physical and statistical parameters are influenced by $\alpha_{1}$ and $\alpha_{2}$ and $D_{1}$ and $D_{2}$ [25, 27, 40]. Thus, these parameters provide detailed information about the adsorption process as described by the two adsorption energy models (Equations 11 and 12).

(11) $$R_{{\mathrm{sat}}_{1}}=\alpha_{1}D_{1}$$

(12) $$R_{{\mathrm{sat}}_{2}}=\alpha_{2}D_{2}$$

From a biological perspective, these parameters illustrate the ability of chimpanzees' and gorillas' OR7D4 olfactory receptors to detect and recognize the pheromone androstenone. As indicated in Table 3, it is observed that $R_{{\mathrm{sat}}_{1}}$ + $R_{{\mathrm{sat}}_{2}}$ (androstenone on chimpanzee OR7D4 receptor) is greater than $R_{{\mathrm{sat}}_{1}}$ (androstenone on gorilla OR7D4 receptor). These modeling results can be explained by the interactions of pheromone molecules with different amino acid residues in each type of binding pocket and across each olfactory system, resulting in distinct olfactory responses at saturation.

#### 3.2.2 Energetic Analysis

##### 3.2.2.1 Half‐Saturation Concentrations $C_{1}$ and $C_{2}$

The energetic parameters $C_{1}$ and $C_{2}$ can provide insights into the energetic characterization of interactions between pheromone molecules and binding pockets in the two analyzed olfactory systems. As evidenced in Figure 3 and Table 3, it is observed that the chimpanzee OR7D4 olfactory receptor binding pockets of Types 1 and 2 exhibit high sensitivity for detecting androstenone molecules, with half‐saturation concentration values of 1.23 × 10^−6^ and 3.60 × 10^−^8mol L^−1^, respectively, compared to the discrimination power of the gorilla OR7D4 olfactory receptor binding pockets (4.14 × 10^−6^ mol L^−1^).

##### 3.2.2.2 Molar Adsorption Energies $\Delta E_{1}$ and $\Delta E_{2}$

The two molar adsorption energies can provide insight into the energetic interactions between the investigated pheromone and the surfaces of the chimpanzee and gorilla olfactory receptors. The one adsorption energy model presented one molar adsorption energy ($\Delta E_{1}$). The two‐adsorption energy model indicated two molar adsorption energies, $\Delta E_{1}$ and $\Delta E_{2}$, corresponding to the first and second types of binding pockets. The molar adsorption energies, $\Delta E_{1}$ and $\Delta E_{2}$, can be calculated using the energetic parameters $C_{1}$ and $C_{2}$, as described in Equations (13) and (14) [41, 42, 43], where R is the ideal gas constant, T is the room temperature (294 K), and $C_{s}$ is the androstenone solubility supposed constant between 294 and 298 K ($C_{s}$ = 1.15 × 10^−5^ mol L^−1^) [44]. Table 4 shows the calculated molar adsorption energies for each olfactory system.

(13) $$\Delta E_{1}=-\mathrm{RT}\ln\left(\frac{C_{1}}{C_{s}}\right)$$

(14) $$\Delta E_{2}=-\mathrm{RT}\ln\left(\frac{C_{2}}{C_{s}}\right)$$

**TABLE 4:** Values of calculated molar adsorption energies $\Delta E_{1}$ and $\Delta E_{2}$.

Energetic parameters	Olfactory systems	
	Chimpanzee OR7D4	Gorilla OR7D4
$C_{1}$ (mol L^−1^)	1.23 × 10^−6^	4.14 × 10^−6^
$C_{2}$ (mol L^−1^)	3.60 × 10^−8^	—
$\Delta E_{1}$(kJ mol^−1^)	5.46	2.49
$\Delta E_{2}$(kJ mol^−1^)	14.09	—

According to the results presented in Table 4, the estimated values of the energies $\Delta E_{i}$ (i = 1, 2) are less than 40 kJ mol^−1^ (ranging from 2.49 to 14.09 kJ mol^−1^). The $\Delta E_{i}$ values indicate that the binding between the pheromone molecules and the OR7D4 olfactory receptors of chimpanzees and gorillas occurs through physical adsorption (physisorption) [25, 27, 40, 45]. Thus, the physical binding mechanism suggests a weak interaction with androstenone molecules, which are easily desorbed. Furthermore, the results indicate that the physical adsorption process is exothermic since the $\Delta E_{i}$ values are positive [27, 46].

The chimpanzee olfactory receptor OR7D4 showed the highest molar adsorption energies in both types of binding pockets, indicating a higher affinity of androstenone for the binding pockets of chimpanzee OR7D4 compared to those of gorillas. These modeling findings can be attributed to the different types of interactions, such as conventional hydrogen bonding, alkyl interactions, carbon‐hydrogen bonding, pi–pi stacking, pi–alkyl, and pi–sigma interactions, between the pheromone and the functional groups of the amino acid residues that compose the studied olfactory receptors [28].

A molecular docking study was performed to elucidate the molecular linkage and interactions of the investigated olfactory systems, as described in the subsequent section.

#### 3.2.3 Molecular Docking Simulation

Molecular docking is a key tool in structural molecular biology and computer‐aided drug design, as it predicts the most likely three‐dimensional conformations of complexes formed by interacting molecules. In this study, iGEMDOCK [47, 48, 49, 50] was used to investigate the interactions between androstenone and OR7D4 receptors from chimpanzees and gorillas. Since no experimental 3D structure of OR7D4 is available in the Protein Data Bank (PDB), the receptor structures were predicted using the homology modeling module integrated in iGEMDOCK, which generates models based on GPCR template structures available in the PDB. This approach is commonly used for GPCRs and olfactory receptors that lack crystallographic structures [51, 52]. Although homology models provide valuable structural information in the absence of experimentally determined receptor structures, they inherently contain structural uncertainties, particularly, in loop regions, side‐chain orientations, and the geometry of the binding pocket. These uncertainties may influence the predicted docking poses and binding scores. Therefore, the docking results should be interpreted as qualitative predictions supporting the proposed ligand‐receptor interaction mechanism rather than definitive quantitative measurements. Nevertheless, homology modeling remains a widely accepted strategy for studying GPCRs and olfactory receptors when experimental structures are unavailable.

The structures were prepared by removing solvent molecules, adding hydrogen atoms in standard positions, and performing energy minimization to optimize their conformations. Docking calculations used the GEMDOCK scoring function, which applies a genetic algorithm to efficiently explore the ligand conformational space within the predefined binding pocket. Ten top‐ranked docking poses were generated and ranked according to their docking scores, and the pose with the best score was selected for subsequent interaction analysis. Although no docking algorithm can guarantee identification of the absolute global minimum‐energy conformation, the adopted search strategy provides a reliable approximation of the most favorable binding mode under the selected docking conditions. Ligand–residue interactions, including van der Waals, alkyl, and C–H bonds, were analyzed and visualized using Discovery Studio [53, 54]. This procedure ensures a clear and reproducible docking methodology.

In docking calculations, the ligand (androstenone) was treated as flexible to allow conformational adjustments, while the protein was kept rigid. The docking procedure targeted the orthosteric binding pocket, identified based on conserved residues and previous studies [12]. Ten poses per receptor were generated, and the best‐scored pose (based on the GEMDOCK scoring function) was selected for detailed analysis. Ligand–residue interactions, including van der Waals, alkyl, and carbon–hydrogen bonding, were evaluated and visualized using Discovery Studio Visualizer [53, 54]. Figure 6 has been updated to include the full receptor structure with the binding pocket clearly highlighted.

For the chimpanzee OR7D4 receptor, molecular docking identified two energetically favorable binding poses of androstenone (*E*_1_ = 6.02 and *E*_2_ = 14.40 [GEMDOCK docking scores, reported in kJ mol^−1^]).

In the best‐scored pose (*E*_1_), the ligand–protein contacts are dominated by van der Waals interactions with residues ARG G:205, ASP G:204, LEU G:263, GLN G:200, SER G:261, and MET G:258, together with an alkyl contact involving VAL G:264 (Table 5).

**TABLE 5:** Different interactions between the androstenone ligand and amino acids of the chimpanzee OR7D4 protein.

Complex	Binding residue (full name)	Types	Distances	Residues solvent accessibility	Interactions
Chimpanzee OR7D4‐androstenone (*E*_1_ = 6.02 kJ mol^−1^)	ARG G: 205	Arginine	3.48	274.99	VDW
	ASP G: 204	Aspartic acid	3.21	—	VDW
	LEU G: 263	Leucine	3.48	39.19	VDW
	GLN G: 200	Glutamine	3.47	—	VDW
	SERG: 261	Serine	3.51	22.77	VDW
	MET G: 258	Methionine	3.78	74.95	VDW
	VALG: 264	Valine	3.25	39.19	Alkyl
Chimpanzee OR7D4‐androstenone (*E*_2_ = 14.40 kJ mol^−1^)	ASP G: 266	Aspartic acid	3.50	105.82	VDW
	VALG: 264	Valine	3.52	40.06	VDW
	LEU G: 257	Leucine	3.53	33.98	Alkyl
	ALA G: 280	Alanine	3.23	137.10	Alkyl
	SER G: 259	Serine	3.53	137.48	C–H bond

In the second best‐scored pose (*E*_2_), interactions include van der Waals contacts and alkyl interactions, with a C–H type contact observed for SER G:259 (Table 5). The three‐dimensional orientations and interaction maps are shown in Figures 4 and 5. Although the docking energy of the lower‐energy pose is relatively low (~6 kJ mol^−1^, relative binding score units), comparable to the thermal energy at room temperature, the olfactory response can still be triggered. This result occurs because multiple weak interactions act cooperatively within the binding pocket, collectively stabilizing the ligand and enabling receptor activation. Such a multimolecular mechanism explains how even short‐lived or weak interactions can generate a detectable olfactory signal.

**FIGURE 4:**
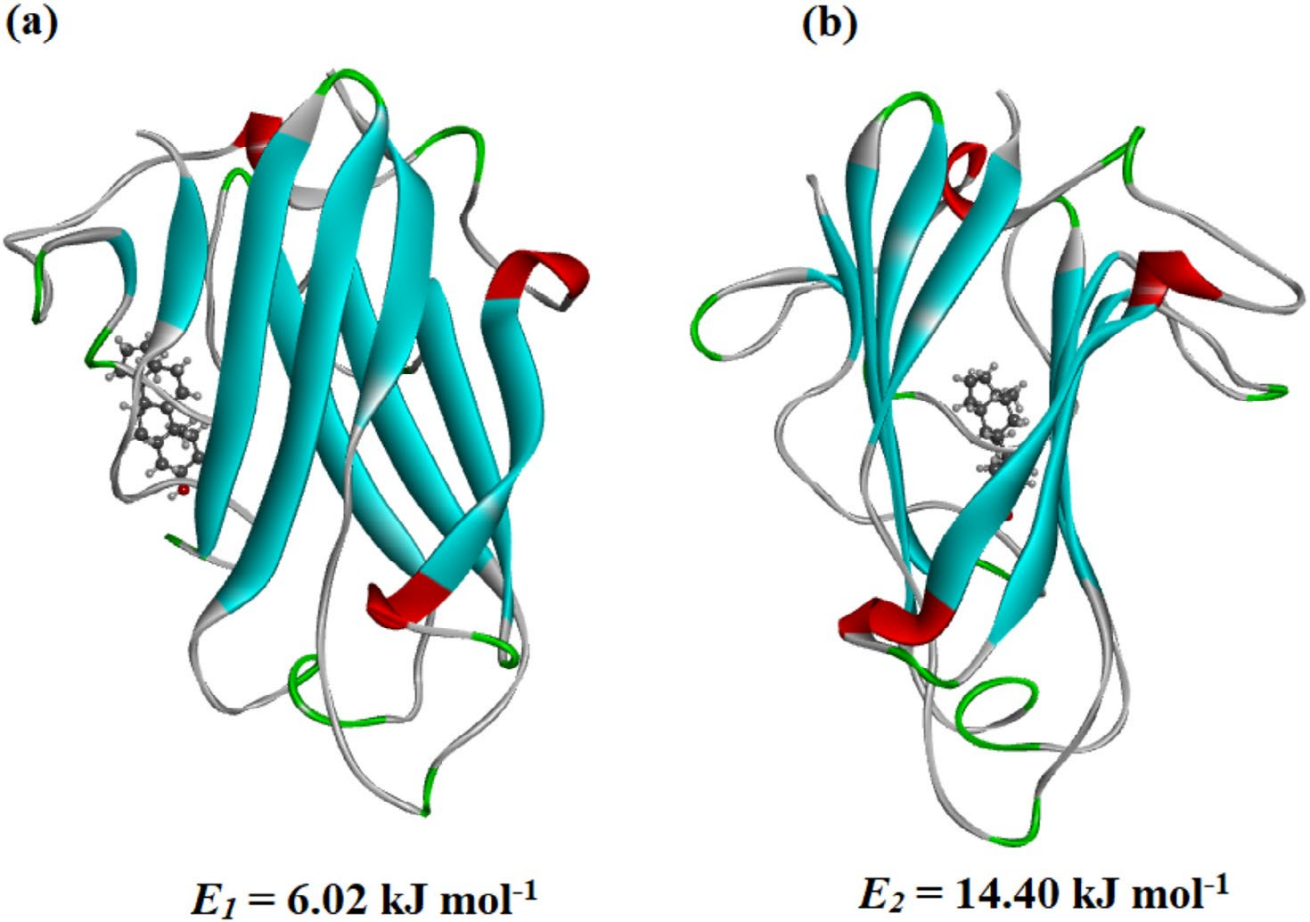
The best poses of chimpanzee OR7D4 binding pocket Type 1 (a) Type 2 (b) androstenone complex.

**FIGURE 5:**
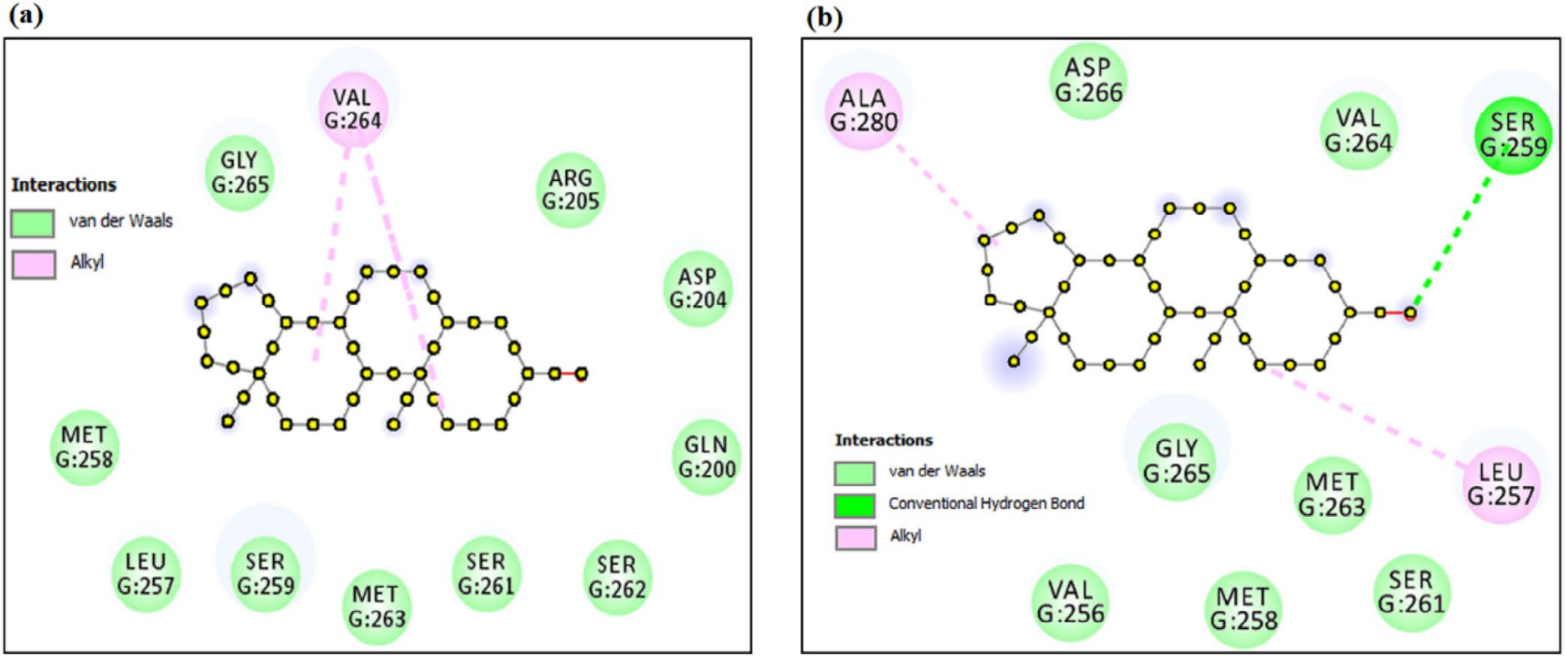
2D of interactions of chimpanzee OR7D4 binding pocket Type 1 (a) Type 2 (b) androstenone complex.

For gorilla OR7D4, visual inspection of the 2D representation (Figure 7) indicates that the ligand forms van der Waals interactions with ARG B:3, PHE B:11, LEU B:12, and alkyl interactions with ILE B:4, ILE B:8, ARG B:7, with distances ranging from 3.01 to 3.52 Å, as detailed in Table 6. Despite high sequence similarity, subtle structural differences between chimpanzee and gorilla OR7D4 sequences may prevent the formation of a second stable binding pose in gorilla. However, an *E*_2_‐like pose is theoretically possible. Our docking results show that the ligand preferentially adopts a single low‐energy orientation in the gorilla receptor.

**TABLE 6:** Different interactions between androstenone and amino acids of gorilla OR7D4.

Complex	Binding residue	Types	Atoms	Distances	Residues solvent accessibility	Interactions
Gorilla OR7D4‐androstenone ($E_{1}$ = 2.12 kJ mol^−1^)	ARG B: 3	Arginine	C	3.45	149.20	VDW
	PHE B: 11	Phenylalanine	C	3.35	170.18	VDW
	LEU B: 12	Leucine	C, O	3.52	161.90	VDW
	ILE B: 4	Isoleucine	CG1‐CD1	3.46	77.96	Alkyl
	ILE B: 8	Isoleucine	C, O	3.01	93.59	Alkyl
	ARG B: 7	Arginine	C	3.47	153.72	Alkyl

These methodological details, including structure preparation, docking flexibility, targeted approach, and interaction analysis, ensure a clear, transparent, and reproducible procedure for investigating ligand–receptor binding.

Concerning the second complex, it was also found that the gorilla OR7D4‐androstenone complex has two best poses (Figure 6) with a significant total energy score ($E_{1}$ = 2.12 kJ mol^−1^). Visual inspection of the 2D representation (Figure 7) of the androstenone pheromone in the binding pockets of Gorilla OR7D4 revealed that carbon atoms may be bonded to the ARG B: 3, PHE B: 11, and LEU B: 12 linker residues via van der Waals interactions, having distances of 3.47, 1.35, and 3.52 Å, respectively. ILE B: 4, ILE B: 8, and ARG B: 7 residues formed alkyl interactions with CG1‐CD1/C, O/C, respectively, with a distance range between 3.46 and 4.01 Å.

**FIGURE 6:**
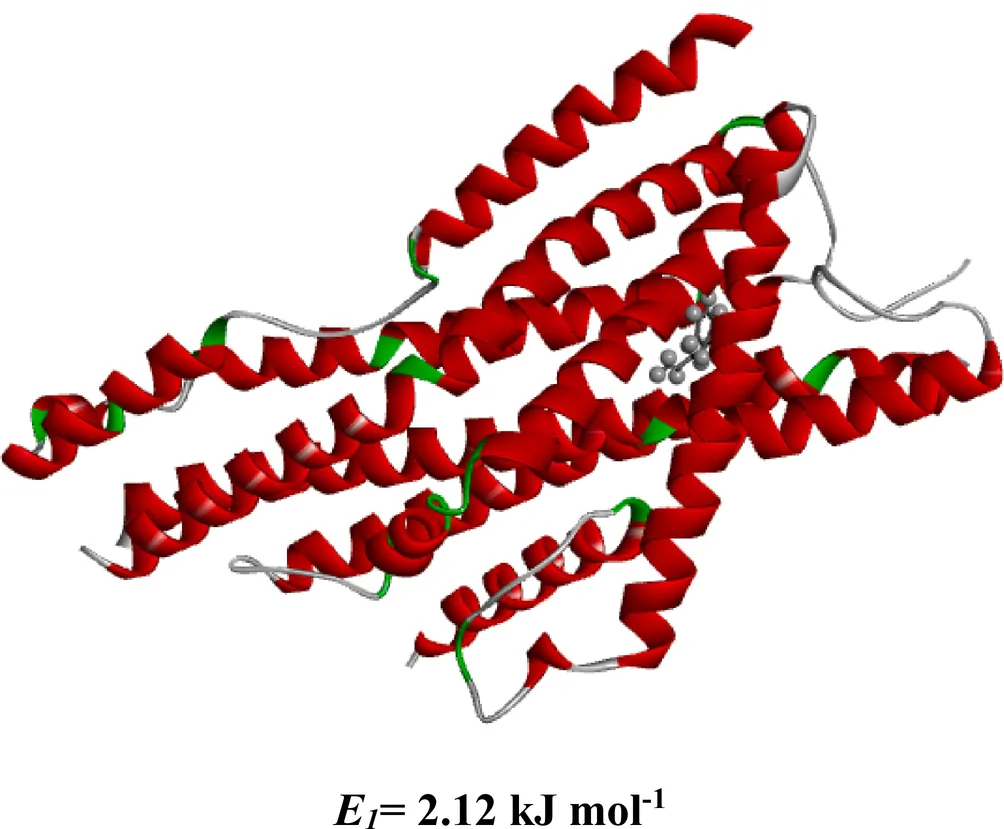
The best pose of gorilla OR7D4 binding pocket‐androstenone complex. *E*_1_ = 2.12 kJ mol^−1^.

**FIGURE 7:**
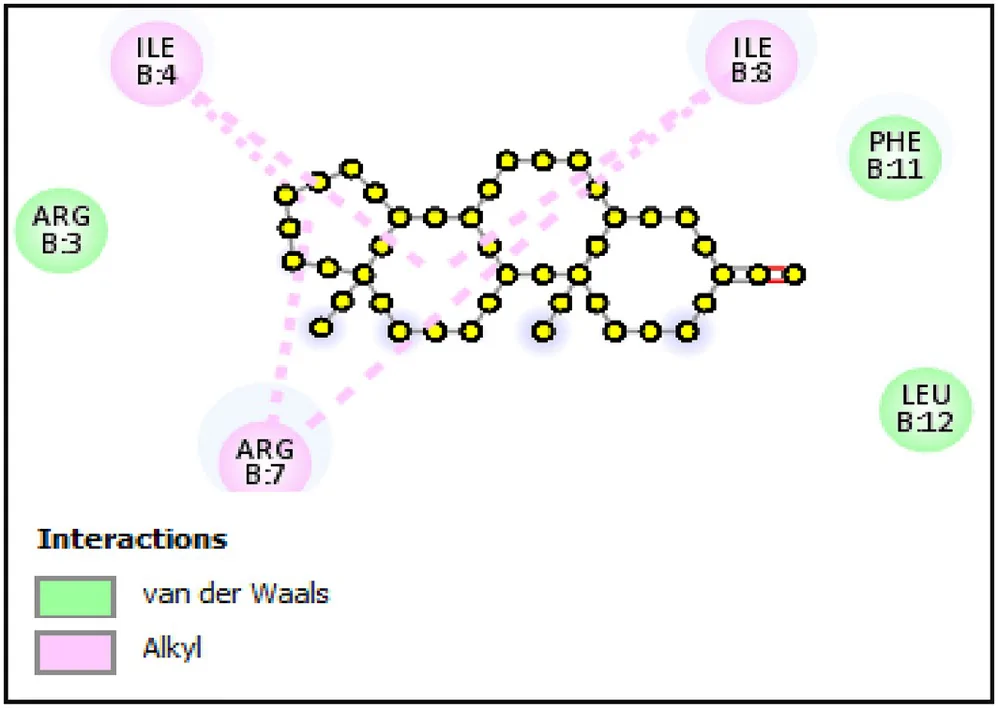
2D of interactions of gorilla OR7D4‐androstenone complex.

#### 3.2.4 Adsorption Entropy

Configurational entropy ($S_{a}$), often called adsorption entropy, provides detailed insights into the order and disorder on the surfaces of chimpanzee and gorilla olfactory receptors during adsorption. The theoretical expression of adsorption entropy can be formulated by Equation (15) [55].

(15) $$\frac{S_{a}}{k_{B}}=-\beta \frac{\partial \ln Z_{\mathrm{gc}}}{\partial \beta }+\ln Z_{\mathrm{gc}}$$

The $S_{a}$ variation in the chimpanzee and gorilla olfactory systems as a function of androstenone concentration is shown in Figure 8A. The entropy profiles for both systems exhibit distinct patterns, attributed to the different models used, with the adsorption entropy being significantly affected by the concentration at half‐saturation. The gorilla olfactory system presents a single peak corresponding to the half‐saturation concentration ($C_{1}$), while the chimpanzee olfactory system shows two peaks corresponding to two half‐saturation concentrations ($C_{1}$ and $C_{2}$). Furthermore, it is observed that the $S_{a}$ increases with androstenone concentration until reaching half‐saturation, at which point the disorder reaches its peak and subsequently decreases. This behavior can be explained by the availability of multiple possibilities for the pheromone to fit into the chimpanzee OR7D4 and gorilla OR7D4 binding pockets at the limiting androstenone concentration. After half‐saturation, the $S_{a}$ decreases as the probability of finding empty pockets decreases, and the surfaces of the investigated olfactory receptors become progressively more occupied until complete saturation is reached.

**FIGURE 8:**
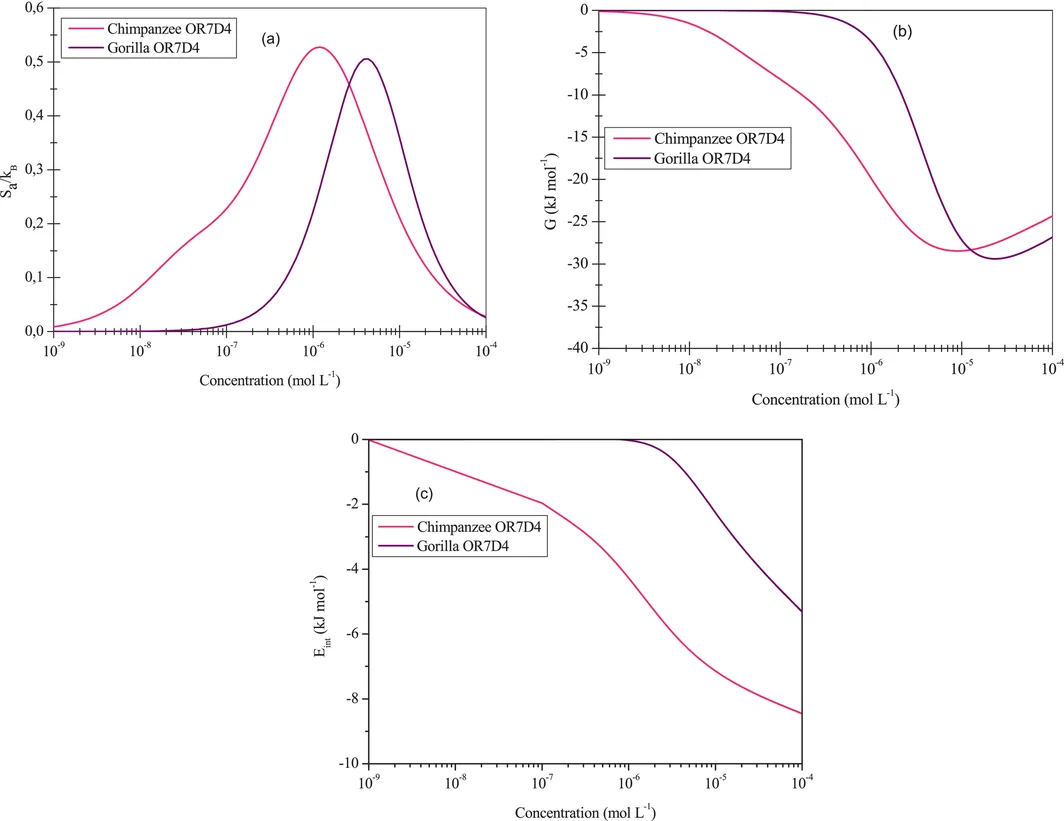
Evolution of $S_{a}$ (a), G (b), and $E_{\mathrm{int}}$ (c) for androstenone on chimpanzee OR7D4 and gorilla OR7D4 at 294 K.

#### 3.2.5 Gibbs Free Energy

Gibbs free energy (G) is used to evaluate the spontaneity of the potential adsorption process in the two olfactory systems. The variation of this thermodynamic function can be analyzed by deriving its theoretical expression based on statistical physics models, according to Equation (16) [55], where the chemical potential (μ) of the olfactory receptor binding pocket may be given by Equation (17).

(16) $$G=\mathrm{n\mu}R_{\text{olfactory}}$$

(17) $$\mu =k_{B}T\ln\left(\frac{C}{Z_{\mathrm{tr}}Z_{\mathrm{vib}}}\right)=k_{B}T\ln\left(\frac{C}{C_{s}}\right)$$

The G variation as a function of androstenone concentration for the olfactory systems of chimpanzees and gorillas is shown in Figure 8b. According to the results, the G values remain consistently below zero, indicating the spontaneity of the adsorption mechanism. At the limiting concentration, the binding pockets of OR7D4 in both chimpanzees and gorillas are practically empty (|G| → 0, with highly negative μ), which favors a robust interaction between the androstenone molecules and the amino acid residues of OR7D4. Thus, the adsorption process occurs spontaneously and intensely, with the G peak values reaching absolute levels at saturation.

#### 3.2.6 Internal Energy

The internal energy ($E_{\mathrm{int}}$), which refers to the total energy contained within an adsorption system, was determined by deriving its theoretical expression using statistical physics models, as shown in Equation (18) [55].

(18) $$E_{\mathrm{int}}=-\frac{\partial \ln Z_{\mathrm{gc}}}{\partial \beta }+\frac{\mu }{\beta }\left(\frac{\partial \ln Z_{\mathrm{gc}}}{\partial \mu }\right)$$

The relationship between $E_{\mathrm{int}}$ and androstenone concentration for the two olfactory systems analyzed is presented in Figure 8C. As shown in Figure 8C, the $E_{\mathrm{int}}$ values present a negative trend, starting from zero and decreasing with increasing androstenone concentration. This behavior suggests that, at the limiting concentration of the adsorption process, the binding pockets of OR7D4 in both chimpanzees and gorillas are predominantly empty, which facilitates an intense interaction between androstenone molecules and the corresponding olfactory receptors. Furthermore, the negative $E_{\mathrm{int}}$ values indicate that the adsorption process associated with olfactory perception is exothermic. The exothermic behavior can be explained by the fact that androstenone molecules release energy during adsorption in mucus.

In summary, Ben Khemis et al. [14, 25, 26, 27, 28, 56, 57, 58] developed a powerful framework based on a sophisticated statistical physics method using advanced adsorption models, which contain physically meaningful physicochemical parameters. These estimated parameters may be applied to microscopically and macroscopically characterize the docking mechanism of various types of odorants, such as pheromones, key food odorants, non key food odorants, and amino acids, on several vertebrate and invertebrate olfactory receptors such as human OR1A1 [14], human OR2W1 [28], mouse mOR‐EG [25], honeybee AcerOr1 [27], zebrafish Z06 [56], horse hoOR8H1 [57], chimpanzee cOR8H1 [57], human hOR8H1 [57], cow btOR1A1 [58], and so forth. Indeed, the utilization of statistical physics models allows for the retrieval of valuable insights into the stereographic and energetic characterizations of olfactory receptor surfaces since direct characterization remains challenging under some circumstances. Therefore, theoretical modeling is applied here as an additional analytical method. It may then be deduced that investigating pheromone molecules or any other odorants may reveal new features to recognize in advance the olfactory properties of the tested olfactory systems during the binding process, which is one of the essential steps in producing the olfactory perception.

## 4 Conclusion

The present study performed a stereographic and energetic analysis of the experimental olfactory dose–response curves of androstenone molecules on chimpanzee and gorilla OR7D4 receptors. To fit the experimental data, two statistical physics models based on the grand canonical ensemble were employed. The fitting results indicate that the two‐energy adsorption model and the one‐energy adsorption model are the most appropriate for predicting the molecular topography and orientation of pheromone molecules on chimpanzee receptor OR7D4 and gorilla receptor OR7D4, respectively. Thus, the simulation results, using statistical physics and molecular fitting theories, demonstrate that:

- Modeling results indicate a nonparallel orientation of androstenone molecules within the chimpanzee OR7D4 and gorilla OR7D4 binding pockets.
- The adsorption process is exothermic and physical since the molar adsorption energies were positive and less than 15 kJ mol^−1^.
- The studied pheromone molecules interact with distinct amino acid residues within each type of binding pocket and olfactory system, allowing the observation of distinct olfactory responses at saturation.
- The results derived from molecular docking calculations agree with those obtained through statistical physics modeling and confirm the energetic findings related to the type and magnitude of interactions between pheromone molecules and the functional groups of amino acid residues present in the studied olfactory receptors.
- The adsorption process occurs spontaneously and exothermically, as indicated by the thermodynamic analysis.

By combining theoretical approaches, statistical physics, and molecular docking simulations, it was possible to understand the molecular‐level mechanism of olfactory adsorption. This aspect remains inaccessible to experimental and empirical methods.

**Nomenclature**
*C*
: concentration at equilibrium in a type *i* binding pocket
*C*_*th*_
: limiting concentration
*C*_*i*_
: half‐saturation concentration
*C*_*s*_
: androstenone solubility
*D*_*i*_
: density of each type of olfactory receptor‐binding pocket
*E*_*i*_
: molar adsorption energy
*E*_int_
: internal energy
*G*
: Gibbs free energy
*n*_*i*_
: number of androstenone molecules anchored in a type *i* binding pocket
*N*_*i*_
: occupation number state of the olfactory receptor binding pockets
$N_{0_{i}}$
: occupancy number of each type of olfactory receptor binding pocket
*R*
: ideal gas constant
*RMSE*
: regression mean square error
$R_{{\text{olfactory}}_{i}}$
: olfactory response of each type of binding pocket
$R_{{\mathrm{sat}}_{i}}$
: olfactory response at saturation relative to each type of binding pocket
*R* ^2^
: determination coefficient
*S*_*a*_
: configurational entropy
*T*
: temperature
*Z*_*i*_
: grand canonical partition function

**Greek symbols**
*α*_*i*_
: coefficient of the transduction relative to $N_{0_{i}}$
β
: factor of Boltzmann
*ε*_*i*_
: adsorption energy of each type of olfactory receptor binding pockets
μ
: chemical potential of olfactory receptor binding pockets

## Author Contributions

**Ismahene Ben Khemis:** conceptualization, investigation, writing – original draft, software, resources. **Ana Carolina Ferreira Piazzi Fuhr:** software, formal analysis. **GuilhermeLuiz Dotto:** conceptualization, writing – review and editing. **Salah Knani:** investigation, validation, funding acquisition, software. **Nizar Lefi:** methodology, visualization, formal analysis, supervision. **Ghadeer Mohsen Albadrani:** validation, methodology, visualization, writing – original draft. **Olfa Noureddine:** investigation, writing – original draft, validation, visualization, formal analysis. **Fatma Aouaini:** conceptualization, resources, investigation. **Abdelmottaleb Ben Lamine:** conceptualization, writing – review and editing.

## Funding

Supporting Project (Grant PNURSP2026R46), Princess NourahbintAbdulrahman University, Riyadh, Saudi Arabia.

## Ethics Statement

The authors have nothing to report.

## Consent

The authors have nothing to report.

## Conflicts of Interest

The authors declare no conflicts of interest.

## Data Availability

The data that support the findings of this study are available from the corresponding author upon reasonable request.
